# Maintenance and Neuronal Cell Differentiation of Neural Stem Cells C17.2 Correlated to Medium Availability Sets Design Criteria in Microfluidic Systems

**DOI:** 10.1371/journal.pone.0109815

**Published:** 2014-10-13

**Authors:** Bu Wang, Sabrina Jedlicka, Xuanhong Cheng

**Affiliations:** 1 Department of Materials Science and Engineering, Lehigh University, Bethlehem, Pennsylvania, United States of America; 2 BioEngineering Program, Lehigh University, Bethlehem, Pennsylvania, United States of America; Temple University School of Medicine, United States of America

## Abstract

**Background:**

Neural stem cells (NSCs) play an important role in developing potential cell-based therapeutics for neurodegenerative disease. Microfluidics has proven a powerful tool in mechanistic studies of NSC differentiation. However, NSCs are prone to differentiate when the nutrients are limited, which occurs unfavorable by fast medium consumption in miniaturized culture environment. For mechanistic studies of NSCs in microfluidics, it is vital that neuronal cell differentiation is triggered by controlled factors only. Thus, we studied the correlation between available cell medium and spontaneous neuronal cell differentiation of C17.2 NSCs in standard culture medium, and proposed the necessary microfluidic design criteria to prevent undesirable cell phenotype changes.

**Methodology/Principal Findings:**

A series of microchannels with specific geometric parameters were designed to provide different amount of medium to the cells over time. A medium factor (*MF*, defined as the volume of stem cell culture medium divided by total number of cells at seeding and number of hours between medium replacement) successfully correlated the amount of medium available to each cell averaged over time to neuronal cell differentiation. *MF* smaller than 8.3×10^4^ µm^3^/cell⋅hour produced significant neuronal cell differentiation marked by cell morphological change and significantly more cells with positive β-tubulin-III and MAP2 staining than the control. When *MF* was equal or greater than 8.3×10^4^ µm^3^/cell⋅hour, minimal spontaneous neuronal cell differentiation happened relative to the control. *MF* had minimal relation with the average neurite length.

**Significance:**

*MF*s can be controlled easily to maintain the stem cell status of C17.2 NSCs or to induce spontaneous neuronal cell differentiation in standard stem cell culture medium. This finding is useful in designing microfluidic culture platforms for controllable NSC maintenance and differentiation. This study also offers insight about consumption rate of serum molecules involved in maintaining the stemness of NSCs.

## Introduction

Neural stem cells (NSCs) have recently attracted significant interest for their promise in treating neurodegenerative disorders, such as Alzheimer’s disease, ischemia and Parkinson’s disease. [1]–[12] Despite progress in neuronal cell differentiation and transplantation of NSCs, future success will require further understanding of the neuronal cell differentiation mechanisms. [2], [4], [5], [7], [9]–[11], [13]–[20] Microfluidics has recently been shown to be a powerful tool in stem cell research, due to the advantage of precise control of individual environmental cues, single cell analysis, real-time measurement and easy integration with electrical stimulation. [21]–[46].

Concentration gradients of cytokine or growth hormone have been created in microfluidic devices to quantitatively study chemical and biological cues that initiate or facilitate neuronal cell differentiation. [22], [47]–[50] Microfluidics have also been used to introduce mechanical or topographical stimulation for the analysis of non-chemical cues on neuronal cell differentiation. [51], [52] The use of microfluidics in NSC research, however, presents an issue with regard to dynamic nutrient concentration. As the culture volume is miniaturized, nutrient consumption from cell metabolism is much more pronounced than conventional bulk culture, while it is well established that NSCs are extremely sensitive to serum depletion. *In vivo* neuronal cell differentiation of NSCs occurs when there is a shortage of blood and oxygen supply, as studied in disease models like ischemia. [4], [15], [16], [53]–[58] For *in vitro* cultures, serum withdrawal is often used to induce neuronal differentiation of NSC. [59]–[61] Based on the available knowledge up to date, we hypothesize that NSCs could undergo neuronal cell differentiation even in the regular NSC culture media if the volume of media available is limited, which after cell metabolism quickly becomes nutrient depleted. While it is desirable to induce differentiation through controlled biological, chemical and physical cues, spontaneous differentiation needs to be characterized to guide microfluidic design and avoid its interference with mechanistic studies.

Here, we used microfluidic devices to control the amount of culture medium available and characterized the phenotype of C17.2 NSCs over three weeks in standard culture medium. C17.2 is an immortalized mouse neural progenitor cell line established by retroviral-mediated transduction of the avian *myc* oncogene into mitotic progenitor cells of neonatal mouse cerebellum, and an important model system in studies of neural regeneration. [9], [11], [12], [59]–[67] C17.2 NSCs have shown the ability to successfully integrate into the central nervous system of animals used as disease models for Parkinson’s, stroke and Alzheimer’s. [9]–[12] Both *in vivo* and *in vitro* studies also demonstrate that C17.2 NSCs undergo neuronal cell differentiation under nutrient depletion, [11], [12], [59]–[61], [64] which makes them an appropriate cellular model for this work. A medium factor (*MF*) was used as a quantitative measure of available medium to each cell per unit time. The *MF* was defined as the volume of culture medium normalized to the total number of cells at seeding and the feeding period. It was controlled using microchannels of various heights, since it is otherwise difficult to reduce the height of culture media to below one millimeter in conventional bulk culture, considering the meniscus. Another strategy to control *MF* was to vary the feeding frequency, with higher frequency making more fresh medium available to each cell over time. Cell morphology and quantified immunocytochemistry results were examined to verify the correlation between the resulting differentiated cell population and the *MF*. Critical thresholds of *MF* to maintain the stem cell characteristics were identified. The range of consumption rate of serum molecules involved in the process is also discussed in the paper.

## Materials and Methods

### Cell culture

Immortalized murine neural progenitor cells C17.2 (established cell line [9], [11], [12], [59]–[67] as a generous gift to the Jedlicka Lab from Dr. Evan Snyder, of the Sanford-Burnham Medical Research Institute) were grown on 100 mm polystyrene tissue culture dishes (BioLite, Fisher Scientific) at 37°C in 5% CO2 in air. The culture medium consisted of high glucose Dulbecco’s modified Eagle medium (DMEM) (HyClone, Fisher Scientific) supplemented with 10% fetal bovine serum (HyClone, Fisher Scientific), 5% horse serum (TCS Biosciences) and 2 mM L-glutamine (MP Biomedicals).

### Microfluidic device fabrication

**Figure 1:**
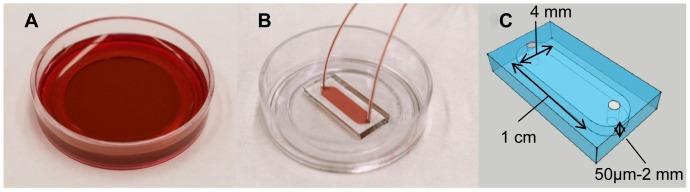
A control and PDMS bonded FluoroDish for cell culture. (A) A standard bulk culture on FluoroDish was used as the control. (B–C) PDMS microchannels with a footprint of 1 cm×4 mm (L×W) and various heights (50 µm, 250 µm, 500 µm, 1 mm and 2 mm) were permanently bonded to FluoroDish to control the amount of medium available to cells. The microchannels were autoclaved prior to bonding and cell culture.

Polydimethylsiloxane (PDMS) microchannels were prepared following the standard soft lithography protocol. Two types of molds were used in this study: SU8 was patterned on silicon wafers for devices with 50 µm and 250 µm heights; micromachined steel molds were used for devices with 500 µm, 1 mm and 2 mm heights. All devices had the same footprint of 1 cm×4 mm (L×W). A 10∶1 mixture of silicone elastomer base and silicone elastomer curing agent (Sylgard 184 silicone elastomer kit, Dow Corning Corporation) was poured onto the molds, degassed, cured at 65–75°C and the microdevices were cut out. Fluid inlets and outlets were drilled using a syringe needle. Microchannels were then autoclaved at 121°C for 1 hour. Afterwards, glass bottomed petri dishes (FluoroDish, World Precision Instruments) and the PDMS microchannels were activated by oxygen plasma, carefully aligned and heated for 5–10 minutes at 65–75°C to produce permanent bonding. The control (a standard microwell culture) and one packaged microfluidic device are shown in Figure 1.

### Cell maintenance and differentiation in microchannels

Trypsinized and suspended C17.2 NSCs were injected into microchannels of different heights: 50 µm, 250 µm, 500 µm, 1 mm and 2 mm. The suspension concentration was adjusted so the surface density was comparable in all devices and was ∼25,000 cells/cm^2^ after initial cell adhesion. After allowing the cells to adhere for 3 hours, the cells were fed periodically every 12, 24, 48 or 96 hours using a syringe pump. Flow rates were used to generate a comparable wall shear stress of 0.004 Pa in all devices. A total of 2.5 times the device volume was injected at every time interval to ensure complete medium replacement. Table 1 summarizes the conditions used. The flow pattern and feeding interval combination was determined by preliminary experiments (Figure S1, Figure S2 and Figure S3). As a control, C17.2 cells were seeded at the same surface density into a FluoroDish without any microchannel. The medium in the control was withdrawn completely and replaced every 48 hours (standard subculture feeding frequency). All devices were kept under humidified environment at 37°C in 5% CO2 in air. Three samples in each condition were immunostained after 1 week, 2 weeks and 3 weeks of culture to monitor the cell phenotype over time. At least five images were captured at random locations from each sample, thus a total of 15 images or more were analyzed under each condition.

**Table 1:** The microchannel geometries and feeding conditions used in this study.

Microchannel height (µm)	Flow rate for cell feeding (µL/hr)	Vol. of medium (µL)	Feeding interval (hour)			
50	250	5	12	24	48	–
250	6,250	25	12	24	48	–
500	25,000	50	12	24	48	–
1000	100,000	100	12	24	48	–
2000	400,000	200	–	24	48	96

### Immunostaining

Cells were fixed in 3.7% paraformaldehyde (Sigma-Aldrich) for 15 min, permeabilized with 0.1% Triton X-100 (Fisher Scientific) in phosphate buffered saline (PBS, Fisher) for 15 min, and blocked by 1% bovine serum albumin (BSA, Sigma-Aldrich) in 0.01% Triton X-100 for 15 min at room temperature. Both primary and secondary antibodies were diluted in 0.1% BSA and 0.001% Triton X-100 solution and then incubated with cells overnight for 8–10 hrs at 4°C. Primary antibodies used were Nestin (clone Rat-401, Fisher Scientific), anti-β-tubulin-III (AlexaFluor488, clone TUJ1, BD Biosciences) and anti-MAP2 antibody (AlexaFluor488, clone AP20, Chemicon). The secondary antibody for Nestin was AlexaFluor546 anti-mouse IgG_1_ (Invitrogen). Finally cell nuclei were stained with 0.002 mg/ml Hoechst No. 33258 (Invitrogen) for 5 min. After rinse, 5 images were captured from each sample using phase-contrast and fluorescence microscopy (Eclipse TE2000U, Nikon). The surface area in each image was 0.15 mm^2^. The average number of β-tubulin-III positive cells per mm^2^ was calculated to characterize the onset of neuronal cell differentiation. MAP2 staining was carried out in selected sets of samples: 50 µm tall microchannel samples under a 48 hr feeding interval, 2 mm tall microchannel samples under a 48 hr feeding interval and fluorodish samples under a 48 hr feeding interval, to confirm the results from β-tubulin-III staining.

### Neurite measurement

The lengths of neurites were measured using immunostained images. Cells with positive staining by β-tubulin-III and neurite outgrowth greater than two times the size of soma were considered as neuronal cells. Neurite outgrowth from each neuronal cell was measured by the NeuronJ plugin in ImageJ (National Institutes of Health). The average number of neuronal cells per mm^2^ was used to characterize neuronal cell differentiation. The average neurite length (total neurite length divided by the number of neurites in an image) was also calculated.

### Medium factor (*MF*)

The *MF* was introduced to quantify the amount of medium available to cells over the feeding period. It was calculated by the following equation:
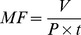
Where *V* is the volume of culture medium, *P* is the total number of cells at seeding, *t* is the time interval between two feeding events.

### Data analysis

All data sets in graphs are presented as average ± standard deviation from repeats in at least three independent devices. When comparing multiple samples in a group, one-way ANOVA test was used with a *p*-value of 0.05. When comparing test samples to the control, two-tailed Student’s t test was used with a *p*-value of 0.05.

## Results and Discussion

### C17.2 differentiation in microchannels with different heights but fixed feeding frequency

First, we examined C17.2 NSCs cultured in microchannels with different heights under fixed feeding frequency. In this case, the *MF* scales linearly with the microchannel height. As a control, cells were seeded at the same surface density in FluoroDish without microchannels (with “c” in all graphs). The average medium height in the open culture is ∼2 mm, calculated by the volume of medium divided by the surface area of the dish bottom, and the cells were fed every 48 hours as in standard subculture protocols.

**Figure 2:**
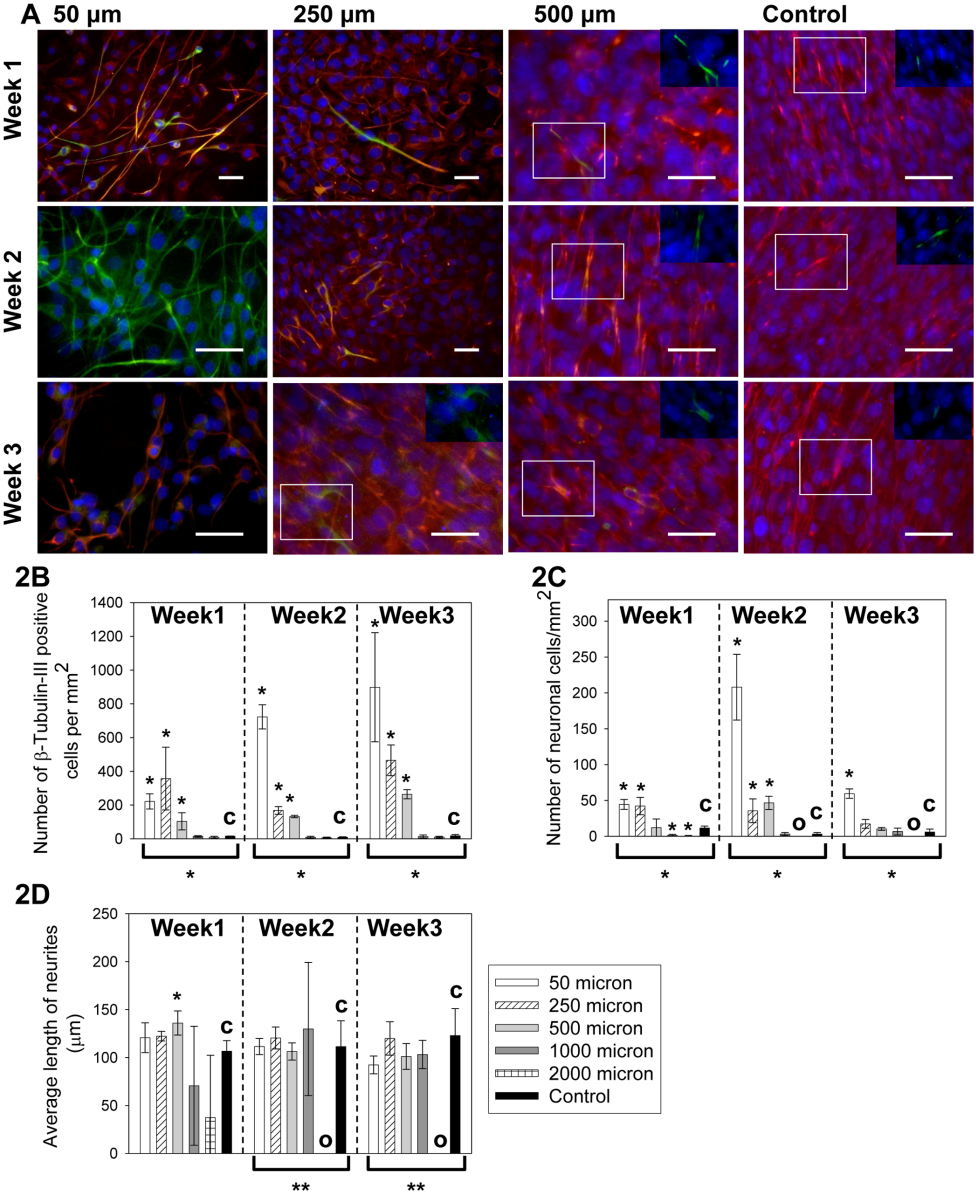
Neuronal cell differentiation of C17.2 cells cultured in microchannels with different heights of 50–2000 µm under a 48 hr feeding interval. (A)The cell morphological change over 3 weeks. Images from the 1000 and 2000 micron samples were not shown since they had similar morphology and staining results as the control for all three weeks. Red: Nestin. Green: β-tubulin-III. Blue: cell nuclei. In some samples, the β-tubulin-III staining was shadowed by the Nestin staining after triple overlay, due to the small number of spontaneously differentiated cells in these samples. In these samples, overlays of the β-tubulin-III and nuclei staining were shown in the inset from the boxed areas. Scale bar = 50 µm. The insets were at the same scale as the main images. (B) The β-tubulin-III positive cell counts per mm^2^ over time. (C) The neuronal cell counts per mm^2^ over time. (D) The average neurite length measurement over time. Bars with “O” in Figure 2C and Figure 2D indicated that no cell was identified as a neuronal cell. In Figures 2B–2D, the control (with “c”) was C17.2 NSCs seeded at the same surface density in FluoroDishes but without microchannels and fed every 48 hours as in standard subculture protocols. Data were shown as mean ± standard deviation. The * above the bars indicated a statistical difference between the sample and the control by two-tailed Student’s t test (*p*<0.05). The */** below the bars indicated a statistical difference in the group by the one-way ANOVA test (*p*<0.05). The ** in Figure 2D indicated a significant difference due exclusively to the samples with no neurite outgrowth. N≥15.

The progression of cell morphology over time is shown in Figure 2A and the quantified immunocytochemistry results are shown in Figure 2B–D. As shown in Figure 2A, groups with lower *MF* values (50 µm, 250 µm and 500 µm microchannels) began to have morphological change consistent with neuronal cell differentiation after 1 week. This trend became dominant after 2 weeks with the 50 µm microchannel yielding almost a pure population of cells with neurite outgrowth and positive β-tubulin-III staining. On the other hand, Nestin staining (red) weakened on the elongated cells, and became nearly undetectable in the 50 um group in week 2, also suggesting neuronal cell differentiation. When the images were analyzed quantitatively, the cell populations in 50 µm, 250 µm and 500 µm microchannels were found to show significantly higher number of β-tubulin-III positive cells compared to the control (with * above the bars, *p* values range from 0.00001 to 0.04) over the entire experimental course of 3 weeks (Figure 2B). The β-tubulin-III positive cell number continued to grow over time, demonstrating an overall tendency towards the neuronal cell differentiation fate. However, neuronal cells (defined here as β-tubulin-III positive cells with neurite length longer than two times that of soma) peaked around 2 weeks and degenerated afterwards (Figure 2A and Figure 2C), as seen by reduction of the neuronal density and weaker β-tubulin-III staining at week 3. The 50 µm microchannel showed significantly higher number of neuronal cells compared to the controls (with * above, *p* values range from 0.0003 to 0.0015) at all time points. The 250 µm microchannel showed significantly higher number of neuronal cells compared to the controls (with * above, *p* values are 0.013 and 0.028 respectively) in the first 2 weeks. The 500 µm microchannel showed significantly more neuronal cells than the control only in week 2 (with * above, *p* = 0.001), but not in week 1 or 3. The 1000 µm and 2000 µm samples, on the other hand, showed comparable or lower level of neuronal cell differentiation compared to the control, as demonstrated by lack of morphological change (images similar to those of the control, not shown) as well as the low number of β-tubulin-III positive cells (Figure 2B) and a baseline presence of neuronal cells (Figure 2C). For the 500 µm microchannel, the average neurite length was slightly higher than the control after 1 week of culture (with * above, *p* = 0.04). The average neurite length (Figure 2D) was the same for the rest of the test conditions and time points (*p* values range from 0.09 to 1), except for those lacking observable neurites (with “O” in Figure 2C and Figure 2D). The low level of neuronal differentiation limits the data points collected from the tall channels (1000 and 2000 µm), resulting in large standard deviation in Figure 2D. Samples from different channel heights but the same weeks were further analyzed using ANOVA. Groups with statistical difference among the samples (*p*<0.05) were marked by brackets and * underneath. For the density of β-tubulin-III positive cells and neurons, the various device heights led to statistically different results at the same time point. For the neurite length comparison, the device height did not create significant difference at week 1. The significant difference of neurite length observed at week 2 and week 3 (with ** underneath, *p*<0.05) was due exclusively to samples with zero neurite outgrowth in the groups. Thus, the neurite length was comparable among all samples with detectable neurite outgrowth at the same week.

### C17.2 differentiation in microchannels with fixed geometry but different feeding frequencies

Next, cell behavior was compared for cultures fed under different frequencies but in microchannels with fixed heights. In this case, the *MF* scales linearly with the reverse of the time interval.

**Figure 3:**
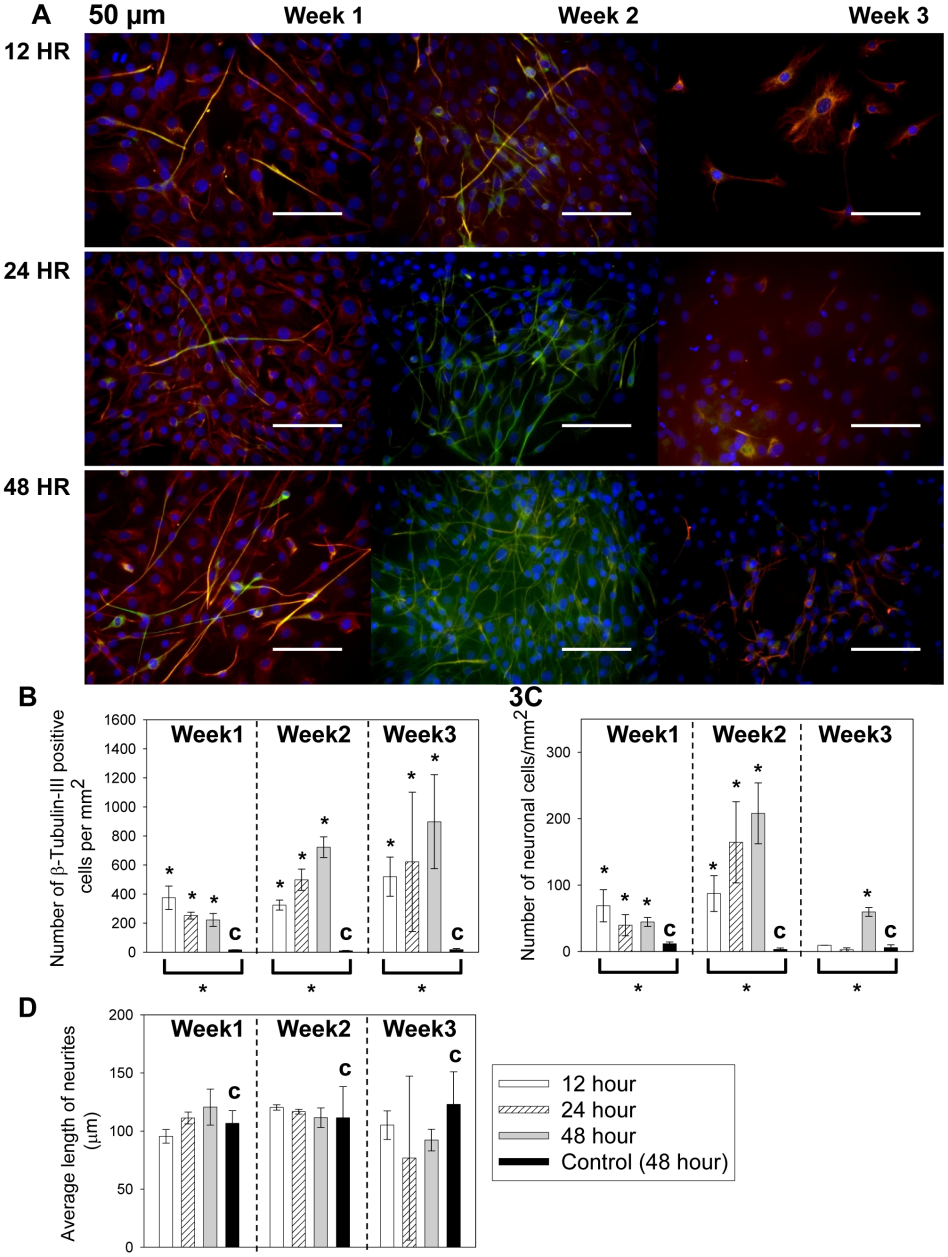
Neuronal cell differentiation of C17.2 cells cultured in 50 µm microchannels with 12, 24 and 48 hour feeding intervals. (A) The cell morphological change over 3 weeks. All groups showed biomarker staining and morphological change consistent with neuronal cell differentiation. Red: Nestin. Green: β-tubulin-III. Blue: cell nuclei. Scale bar = 100 µm. (B) The β-tubulin-III positive cell counts per mm^2^ over time. (C) The neuronal cell counts per mm^2^ over time. (D) The average neurite length measurement over time. In Figures 3B–3D, the control (with “c”) was C17.2 NSCs seeded at the same surface density in FluoroDishes but without microchannels and fed every 48 hours as in standard subculture protocols. Data were shown as mean ± standard deviation. The * above the bars indicated a statistical difference between the sample and the control by two-tailed Student’s t test (*p*<0.05). The * below the bars indicated a statistical difference in the group by one way ANOVA (*p*<0.05). N≥15.

The morphological change of cells in 50 µm microchannel over time is shown in Figure 3A and the quantified immunocytochemistry results are shown in Figure 3B–D. In all the groups, cells began to develop smaller cell bodies and neurite outgrowth after 1 week (Figure 3A). After 2 weeks more cells showed neural morphology and positive β-tubulin-III staining. At the same time, the β-tubulin-III staining became stronger as Nestin staining weakened. After 3 weeks of culture, the cells with long neurites started to deteriorate. Instead, another population with flattened cell body and relatively short and unbranched processes began to dominate, which co-expressed both Nestin and β-tubulin-III. As shown in Figure 3B, the cell populations in 12 hr, 24 hr and 48 hr groups all showed significantly higher number of β-tubulin-III positive cells than the control in all 3 weeks (with * above, *p* values range from 0.00006 to 0.009). The β-tubulin-III positive cell number increased constantly over time, indicating a steady population growth in the overall neuronal cell differentiation path. The large error bars for samples in week 3 are caused by non-uniformly distributed cell population mixture associated with neuronal degeneration. The numbers of neuronal cells are shown in Figure 3C. The initial growth, peak growth and deterioration of neuronal cells were observed in all groups. At initiation and peak stages, all groups showed significantly higher number of neuronal cells compared to the control (with * above, *p* values range from 0.002 to 0.04). At week 3, the difference in neuronal cell number between the microfluidic groups and the control diminished, except for the group with lowest *MF* (with * above, 48 hours, *p* = 0.0003). The average neurite length was comparable for all groups (*p* values range from 0.15 to 1) as shown in Figure 3D. The large standard deviation of neurite length at week 3 is a result of neuronal degeneration, leaving behind a mixed population of cells with broad distribution of neurite length. The ANOVA test of different feeding conditions at each time point indicated that the feeding intervals significantly impacted the density of β-tubulin-III positive cells and neurons in the 50 µm channels (brackets and * underneath the groups, *p*<0.05), but not the neurite length (*p*>0.05).

**Figure 4:**
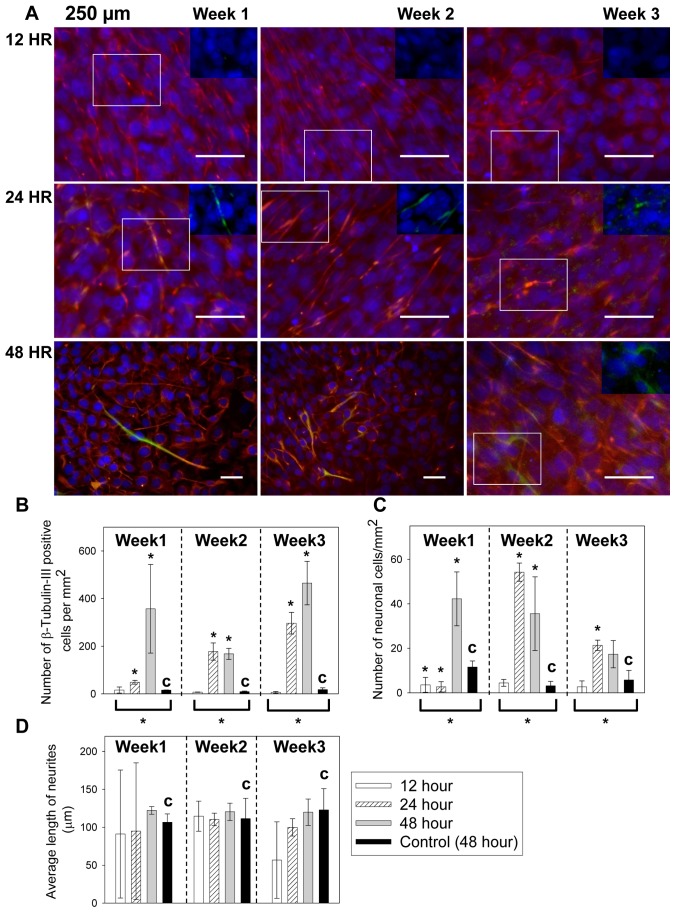
Neuronal cell differentiation of C17.2 cells cultured in 250 µm microchannels with 12, 24 and 48 hour feeding intervals. (A) The cell morphological change over 3 weeks. To better demonstrate the positive β-tubulin-III staining in the 24 hour and 48 hour samples, overlays of the β-tubulin-III and nuclei staining were shown in the inset from the boxed areas. In comparison, insets of β-tubulin-III and nuclei staining were also shown for the 12 hour samples, but β-tubulin-III staining was not visible. Red: Nestin. Green: β-tubulin-III. Blue: cell nuclei. Scale bar = 50 µm. The insets were from the boxed region and were at the same scale as the main images. (B) The β-tubulin-III positive cell counts per mm^2^ over time. (C) The neuronal cell counts per mm^2^ over time. (D) The average neurite length per mm^2^ over time. In Figures 4B-4D, the control (with “c”) was C17.2 NSCs seeded at the same surface density in FluoroDishes but without microchannels and fed every 48 hours as in standard subculture protocols. Data were shown as mean ± standard deviation. The * above the bars indicated a statistical difference between the sample and the control by two-tailed Student’s t test (*p*<0.05). The * below the bars indicated a statistical difference in the group by one way ANOVA (*p*<0.05). N≥15.

The morphology change of cells over time in 250 µm microchannel is shown in Figure 4A and the quantified immunocytochemistry results are shown in Figure 4B–D. The cell populations in 24 hr and 48 hr groups showed neurite outgrowth (Figure 4A) and significantly higher number (Figure 4B) of β-tubulin-III positive cells than the control at all time points (with * above the bars, *p* values range from 0.0003 to 0.03). However, the 12 hr group had minimal morphological change (Figure 4A) over 3 weeks. The numbers of β-tubulin-III positive cells in the 12 hr group were comparable to the control at all stages (*p* values range from 0.07 to 1). The numbers of neuronal cells are shown in Figure 4C. The initial growth, peak growth and deterioration of neuronal cells were observed in 24 hr and 48 hr groups. At the peak stage, groups with lower *MF* values, i.e. with 24 hr and 48 hr feeding intervals, showed significantly higher number of neuronal cells than the control (*p* = 0.00004 and 0.028 respectively). The 12 hr group with higher *MF* started with significantly lower number of neuronal cells comparing to the control (with * above, *p* = 0.03), but the difference became insignificant later (*p* = 0.4 at 2 weeks and *p* = 0.3 at 3 weeks). The average neurite length was comparable to the control for all the samples (*p* values range from 0.09 to 0.9) as shown in Figure 4D. The ANOVA test for the 250 µm samples at each week also indicated that the density of β-tubulin-III positive cells and neurons changed significantly with the feeding intervals (brackets and * underneath the groups, *p*<0.05), but not the neurite length.

**Figure 5:**
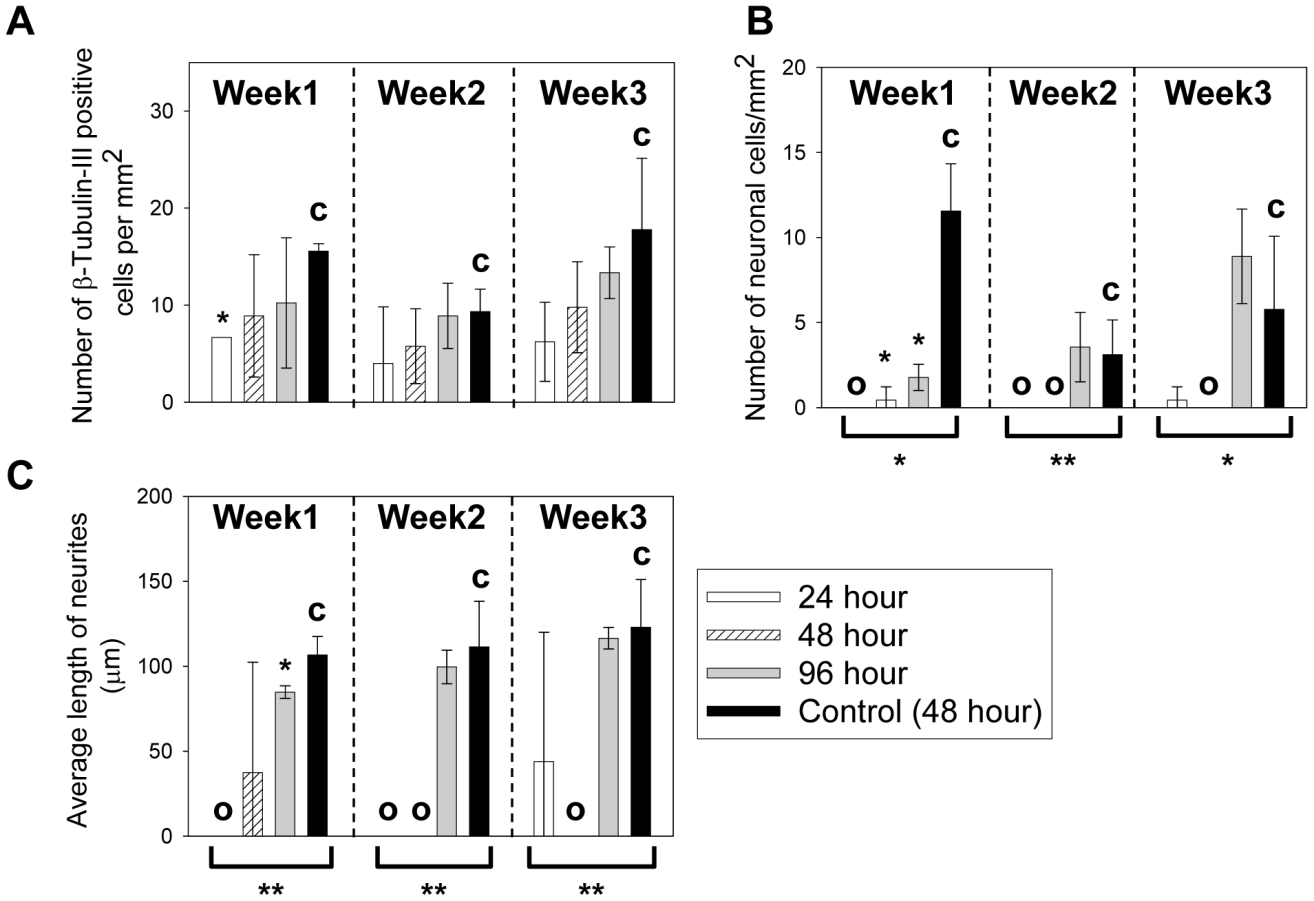
Behavior of C17.2 cells cultured in 2000 µm microchannels with 24, 48 and 96 hour feeding intervals. (A) The β-tubulin-III positive cell counts per mm^2^ over time. (B) The neuronal cell counts per mm^2^ over time. (C) The average neurite length per mm^2^ over time. Groups lacking neuronal cells were labeled with “O” in Figure 5B and Figure 5C. The control (with “c”) was C17.2 NSCs seeded at the same surface density in FluoroDishes but without microchannels and fed every 48 hours as in standard subculture protocols. The * above the bars indicated a statistical difference between the sample and the control by two-tailed Student’s t test (*p*<0.05). The * below the bars indicated a statistical difference in the group by one way ANOVA (*p*<0.05). The ** in Figure 5C indicates a significant difference due exclusively to the samples with no neurite outgrowth. N≥15.

To ensure that the increase in neuronal cell differentiation observed in microchannels were due to the nutrient concentration but not PDMS or feeding under a flow condition, cell population in 2000 µm microchannels was compared to that in bulk culture (Figure 5), as the *MF* factors were similar in these cases. Little morphological and biomarker change was observed over time in all samples (images not shown), demonstrating minimal neuronal cell differentiation. When the results were quantitatively analyzed, the cell population with the 24 hr feeding intervals showed lower number of β-tubulin-III positive cells compared to the control (Figure 5A) at 1 week (with * above, *p* = 0.00004). This is possibly due to more thorough medium replacement in microfluidics as compared to traditional pipetting in petri dishes: the channel is flushed with fresh medium 2.5 times the channel volume at every cell feeding, while in traditional petri dish culture, the old medium is suctioned up and replaced with equal volume of fresh medium once. The rest of the microfluidic samples shared comparable number of β-tubulin-III positive cells to the control (*p* values range from 0.08 to 0.9). As shown in Figure 5B, all samples had lower number of neuronal cells compared to the control in the first week (*p* values range from 0.02 to 0.04), but the difference diminished later. From Figure 5C, it was observed that the average neurite length of most samples were similar to the control except for those without noticeable neurite outgrowth (with “O” in Figure 5B and Figure 5C). The ANOVA tests for samples from the same time points demonstrated comparable density of β-tubulin-III cells and neurite length in the 2000 µm channels. The density of neuronal cells were significantly different under the various feeding intervals (brackets and * underneath the groups, *p*<0.05), but the difference was due to a reduced neuronal density than the control. The neurite length was comparable The ** under the groups in Figure 5C and 5D indicate difference due exclusively to the samples with zero positive cells. These results support that the enhanced neuronal cell differentiation associated with low channel heights and long feeding intervals is a result of nutrient consumption, not PDMS or the flow feeding.

### Confirmation of Differentiation by MAP2 staining

**Figure 6:**
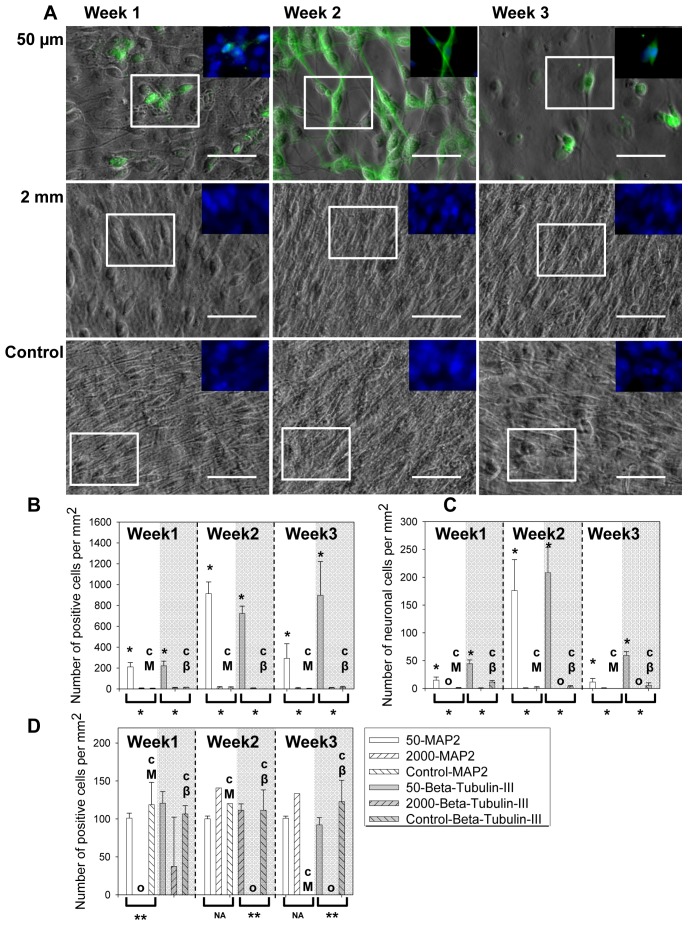
C17.2 cells behavior characterized by MAP2 staining. (A) The morphology of cells shown as overlaid phase contrast and MAP2 staining images. Green: MAP2. Blue: cell nuclei. Scale bar = 50 µm. The insets of MAP2 and cell nuclei staining were from the boxed region and were at the same scale as the main images. (B) MAP2 and β-tubulin-III (gray shades) positive cell counts per mm^2^ over three weeks. Both staining showed similar trend of cell differentiation versus test conditions. (C) The neuronal cell count per mm^2^ over time determined by cell morphology plus MAP2 or β-tubulin-III (gray shades) staining. (D) The average neurite length per mm^2^ over time with positive MAP-2 or β-tubulin-III (gray shades) staining. The controls (with “cM” for MAP2 control and “cβ” for β-tubulin-III control) were C17.2 NSCs seeded at the same surface density in FluoroDishes but without microchannels and fed every 48 hours as in standard subculture protocols. Bars with “O” in Figure 6C and Figure 6D indicated that no cell was identified as a neuronal cell. Data were shown as mean ± standard deviation. The * above the bars indicated a statistical difference between the sample and the control by two-tailed Student’s t test (*p*<0.05). The */** below the bars indicated a statistical difference in the group by the one-way ANOVA test (*p*<0.05). The ** in Figure 6D indicated a significant difference due exclusively to the samples with no neurite outgrowth. The groups labeled with NA below the brackets did not have enough number of neurites for ANOVA analysis. N≥15.

MAP2 staining was carried out in selected samples to confirm the differentiation characterization by β-tubulin-III staining. Here a MAP2 antibody targeting both MAP2a (expressed constitutively in neuronal cells) and MAP2b (expressed postnatal) isoforms was selected, so it stained differentiated neuronal cells at similar stages as the β-tubulin-III antibody. The selected culture conditions are: 1) a condition in microchannels with the highest level of differentiation, i.e. 50 µm tall microchannel samples under a 48 hr feeding interval, 2) a condition in microchannels with *MF* equivalent to that of the control, i.e. 2 mm tall microchannel samples under a 48 hr feeding interval and 3) the open culture control, i.e. fluorodish samples under a 48 hr feeding interval. The morphology of the cells is shown in Figure 6A with MAP2 staining (green) overlaid with the phase contrast images. The insets show the overlaid MAP2 and nuclei staining. MAP2 staining was obvious only in the 50 µm samples, but not the other two. The expression of MAP2 peaked at week 2 and decreased at week 3.

The quantitative analysis of MAP2 staining is shown in Figure 6B-6D together with that of the β-tubulin-III staining. The 50 µm microchannel samples demonstrated higher level of differentiation than the control (Figure 6B) marked by MAP2 and β-tubulin-III expression (with * above, *p* range from 0.0001 to 0.007) while both biomarkers demonstrated cells in the 2000 µm microchannels to be comparable to the control (*p* range from 0.66 to 0.96). When neuronal cells were counted by positive MAP2 staining and neurite outgrowth greater than twice of the cell body, the dependence of neuronal cell density on the culture condition were comparable to the findings from β-tubulin-III staining (Figure 6C). MAP2 staining showed initiation, peak and degeneration of neuronal differentiation in the 50 µm microchannels similar to β-tubulin-III staining. The neurite length determined from MAP2 positive cells was found to be mostly comparable to the control at the same week, consistent with the results from β-tubulin-III staining (Figure 6D). However, the number of neurites in the MAP2 stained samples was not large enough to carry out ANOVA analysis (groups labeled with NA below brackets). The comparable staining results of MAP2 and β-tubulin-III support our method of identifying differentiated neuronal cells by β-tubulin-III staining.

### Correlation of the *MF* to neuronal cell differentiation

To quantify the average amount of medium available to cells over time, the *MF* was introduced, which is the amount of culture medium available to each cell (cell number from initial seeding) divided by the feeding interval.

**Figure 7:**
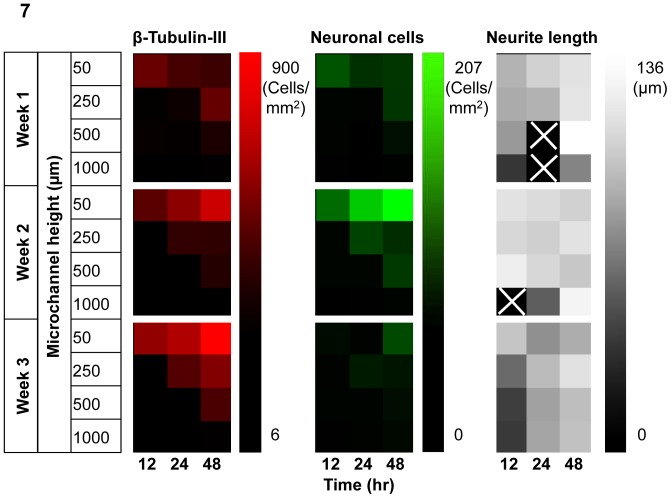
Summary of cell phenotypes with microchannel heights of 50 µm to 1000 µm and feeding frequencies of 12 hours to 48 hours. Groups with small *MF*s (towards top right corner of each graph) generally had more obvious neuronal cell differentiation as demonstrated by brighter colors representing higher density of cells with positive β-tubulin-III staining and neural morphology (first and second columns). The neurite length (third column), however, did not seem to have a strong correlation with the *MF*. The samples without noticeable neurite outgrowth were marked with a cross sign.

As summarized in the heat maps in Figure 7, experiment groups with low *MF*s (top right corner of each graph) were often associated with more prominent neuronal cell differentiation marked by more cells with positive β-tubulin-III staining (red) and more neuronal cells (green) than groups with high *MF*s (lower left corner of each graph). The neurite length is mostly ∼100 µm except for those without observable neurite outgrowth (marked with a cross in the last panel of Figure 7).

**Figure 8:**
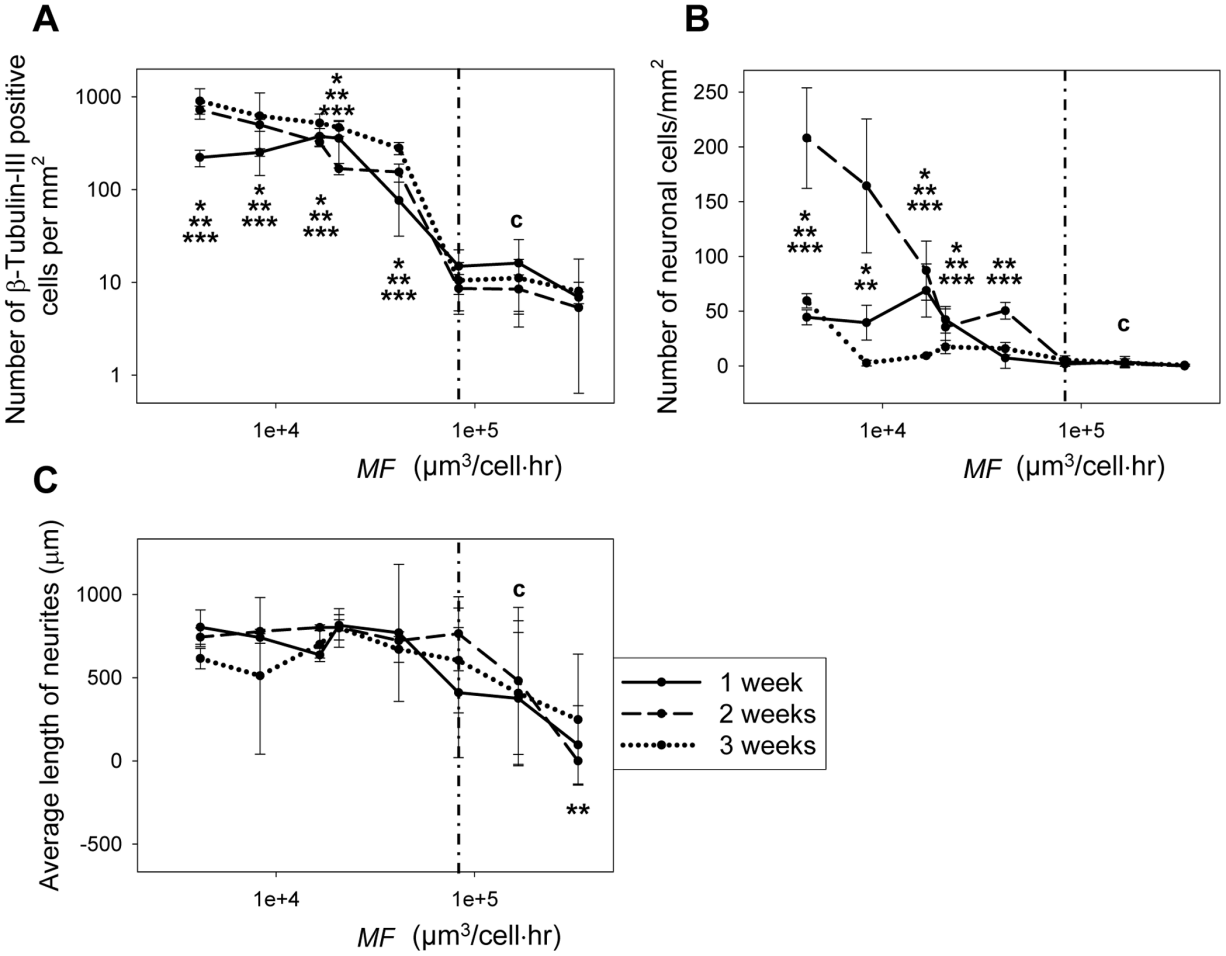
Correlation between *MF* and neuronal cell differentiation. (A) Samples with *MF* number smaller than 8.3×10^4^ µm^3^/cell⋅hour had significantly higher numbers of β-tubulin-III positive cells than that of the control. Samples with *MF* numbers equal to or larger than 8.3×10^4^ µm^3^/cell⋅hour had similar β-tubulin-III positive cell population compared to the control. (B) Samples with *MF*s smaller than 8.3×10^4^ µm^3^/cell⋅hour generally had significantly higher numbers of neuronal cells while samples with *MF*s equal to or larger than 8.3×10^4^ µm^3^/cell⋅hour were mostly comparable to the control. (C) The average neurite length had minimal correlation with the *MF*. The control (with “c”) was C17.2 NSCs seeded at the same surface density in FluoroDishes but without microchannels and fed every 48 hours as in standard subculture protocols. In all data points, N≥15. * indicates statistical difference compared to the control after 1 week, ** for 2 weeks and *** for 3 weeks (p<0.05). The vertical dash line represents the critical *MF* of 8.3×10^4^ µm^3^/cell⋅hour.

Statistical analysis was carried out to quantitatively investigate the correlation between *MF* and neuronal cell differentiation. *MF* values of all experiment groups were calculated and data sets were re-organized based on their *MF* values. As shown from the relatively tight error bars in most data points in Figure 8, the parameter *MF* is the dictating factor that controls the behavior of C17.2 in standard culture media. The critical *MF* of 8.3×10^4^ µm^3^/cell⋅hr is indicated by the vertical dash line in Figure 8. The control is labeled with “c”. Demonstrated in Figure 8A, samples with *MF* smaller than 8.3×10^4^ µm^3^/cell⋅hr had significantly larger numbers of β-tubulin-III positive cells than that of the control (*p* values range from 7×10^−15^ to 0.0004). Samples with *MF* numbers equal or greater than 8.3×10^4^ µm^3^/cell⋅hr had similar β-tubulin-III positive cell population compared to the control (*p* values range from 0.1 to 0.8). This correlation held true over the entire experiment course of 3 weeks (* indicates statistical difference compared to the control after 1 week, ** for 2 weeks and *** for 3 weeks). The number of neuronal cells also correlated well with the *MF* (Figure 8B). Samples with *MF*s smaller than 8.3×10^4^ µm^3^/cell⋅hr generally had significantly higher numbers of neuronal cells (*p* range from 5×10^−13^ to 0.013) while samples with *MF* equal to or greater than 8.3×10^4^ µm^3^/cell⋅hr were comparable (*p* values range from 0.1 to 1) to the control. Out of 24 data sets, only 2 fell out of this correlation, i.e. at *MF* = 41,500 µm^3^/cell⋅hr at week 1 (*p* = 0.28) and *MF* = 8,300 µm^3^/cell⋅hr at week 3 (*p* = 0.9). These two outliers were likely a result of time-dependent uprising and degeneration of the neuronal cell population over time. When further examining the morphological change of cells over time, an almost pure neuronal cell population of high density was observed at week two for groups with *MF* of 8.3×10^3^ µm^3^/cell⋅hr or smaller. On the other hand, the average neurite length mostly remained unchanged with the *MF* (Figure 8C) regardless of the level of spontaneous differentiation (Figure 2–6). Thus, the *MF* is a parameter that can be used to predict spontaneous neuronal cell differentiation in standard culture medium for C17.2 NSCs, while the neurite length is not strongly controlled by the *MF*.

While the microfluidic culture introduced a few differences from conventional culture, including the exposure to shear stress, presence of PDMS and rate of nutrient depletion, the nutrient availability seemed to be the main contributor to the observed spontaneous differentiation. In our preliminary studies (Figure S1, Figure S2 and Figure S3), we demonstrated that leachant from PDMS, if present, did not induce higher C17.2 differentiation than the control of conventional culture. In addition, the optimal shear stress to maintain C17.2 NSCs was found to be around 0.004 Pa. This shear stress is within the range normally experienced by NSCs *in vivo*. [65] NSCs in their natural physiological environment experience shear stress generated by interstitial flow, which falls into the range of 0.01 to 0.001Pa. [65]–[70] Thus the flow condition used in the study is not expected to be detrimental to the NSCs. C17.2 cells cultured in the 2 mm-thick channel showed comparable or even lower spontaneous differentiation when compared to the conventional static culture (Figure 5), indicating the shear condition used here did not contribute to the enhanced differentiation in the thinner channels. Thus, the correlation of the cell phenotype with the *MF* demonstrates that *MF* is the major contributor to the observed high levels of spontaneous differentiation in thin devices or long feeding intervals.

Our observation of spontaneous neuronal cell differentiation under low *MF* is consistent with reports in the literature from *in vivo* studies: in response to nutrient depletion and the resulting damage in the neural network, NSCs go through neuronal cell differentiation in an effort to repair the damage. [4], [15], [16], [53]–[58] Nutrient depletion by serum withdrawal is also the predominant method to induce neuronal differentiation of C17.2 NSCs *in vitro*. [59]–[61], [64], [71].

Although it is difficult to predict or determine the molecular source that contributes to the process, the observed critical *MF* suggests that the key molecules should have been consumed and reached a critically low concentration to induce spontaneous differentiation. For example, epidermal growth factor (EGF) may be an important player in neuronal cell differentiation: stem cell culture medium contains 10 ng/ml EGF while differentiation medium contains no EGF. [13], [14], [74]–[77] Based on the *MF* threshold, the consumption rate of EGF is predicted to be greater than 3.5×10^−23^ mol/s⋅cell. This prediction is consistent with experimental measurements that places consumption rate of EGF at 3.4×10^−22^ mol/s⋅cell for a fast EGF consuming cell model A431 epidermoid carcinoma cells. [78] Thus, the *MF* threshold identified here may offer insight about the consumption rates of key chemicals involved in neuronal cell differentiation.

As observed in all the samples showing NSC differentiation, the neuronal cell population experienced a dynamic process of growth, peak and degeneration over the 3 week culture period. Although neurons are terminally differentiated cells and cannot proliferate, [1], [79]–[82] primary neurons are capable of surviving *in vitro* culture for weeks. [48], [49], [83] The fast degeneration of the spontaneously differentiated neuronal cells in the case described herein may be caused by two possible reasons: an unfavorable environment to maintain neuronal cells and lack of an integrated neuronal cell network that is often required for long-term neuronal survival *in vitro*. The culture medium and feeding pattern was optimized for culturing NSCs instead of neuronal cells. Although neuronal cells can differentiate spontaneously from NSCs through nutrient consumption, maintaining them might require medium adjustment as neural apoptosis can be induced by a variety of stimuli such as growth factor concentration change and glucose concentration change. [84]–[93] Additionally, neurons are much more sensitive to shear stress than NSCs, thus cell feeding through laminar flow may damage the neuronal cells. [48], [49], [77], [94]–[99] The other possible cause is immaturity of neural network. The protocols to induce NSC differentiation (C17.2 cells and others) and maintain the resulting neuronal cells usually require chemical stimuli concurrent with nutrient depletion. [4], [12], [59]–[62], [64], [73], [75] These stimuli promote the formation of dopaminergic neurons and neural communication, which are key to their integration in certain areas of the brain. [2]–[5], [11], [12], [16], [81] The neuronal cells formed in this study by nutrient consumption alone may lack the proper cues to integrate into a network, thus degenerate soon after differentiation.

While the cell population and nutrient availability are widely different in this study, the neurite outgrowth length appears consistent in majority of the test conditions. It has been reported that when using the same differentiation method, the length of neurites is characteristic of the physical environment. Curley et al. have reported characteristic neurite length of differentiated C17.2 NSCs on materials of different elasticity. [100] Yang et al. have used complex environmental physical cues to control both the neurite length and orientation of differentiated C17.2 NSCs. [101] The consistency of neurite outgrowth length observed in this study is likely a result of comparable physical exposures in all microfluidic samples, while the biochemical cues from nutrient restriction don’t seem to play a key in neurite development after initiating NSC differentiation.

Despite the observed degeneration of differentiated neuronal cells, the β-tubulin-III positive cell population generally maintained a steady growth in our study, even after neuronal degeneration. This was a result of an emerging cell population with flattened morphology and multiple radially extended unbranched short processes in week 3. These morphological characteristics are consistent with those of intermediate cell types between NSCs and neurons. [102], [103] While β-tubulin-III is often used an early neural marker, [104]–[106] any neuron-restricted progenitor cell type between the NSC phase and differentiated post-mitotic neuron phase could express β-tubulin-III. [107]–[109] The continuous proliferation of the intermediate cells kept the β-tubulin-III positive cell on the rise, [76], [102], [110]–[114] however, they were incapable of replacing the degenerated neuronal cells in the culture environment studied here. Our observation suggests different signals and culture environments may be required for the branched differentiation pathways from C17.2 NSCs to neuronal cells and from intermediate cells to neuronal cells.

## Conclusions

The *MF* successfully predicts the outcome of C17.2 NSCs in standard culture medium. The *MF* smaller than 8.3×10^4^ µm^3^/cell⋅hour causes spontaneous neuronal cell differentiation marked by a higher density of cells with positive β-tubulin-III or MAP2 staining and neural morphology than the control. On the other hand, minimal spontaneous neuronal cell differentiation is observed when the *MF* is equal to or larger than 8.3×10^4^ µm^3^/cell⋅hour. The average neurite length does not have a strong correlation with the *MF*. The *MF* can be controlled by several experimental factors such as cell density, cell medium volume and feeding time interval to maintain the stem cell status of C17.2 NSCs or to induce various levels of neuronal cell differentiation. Thus, the findings offer guidelines to microfluidic system design for controllable NSC maintenance and differentiation.

## Supporting Information

Figure S1 **Cell adhesion and proliferation in 50 µm microchannel with continuous flow of regular stem cell culture medium.** C17.2 cell adhesion and proliferation was tested in 50 µm tall microchannel with continuous flow of 1, 2, 3 and 4 µL/hour. (S1.A–S1.D) Cell morphologies after 1 day of continuous flow of medium at different flow rates. (S1.E) The surface area covered by cells after 1 day of culture was normalized to that after 3 hour of static adhesion (S1.E) to estimate the number of cells in the microchannels. Continuous medium feeding at 3 µL/hour yielded the highest number of adherent cells after 1 day of continuous flow, but little cell proliferation was observed. Scale bar = 100 µm. N≥15.
(TIF)

Figure S2 **Cell adhesion and proliferation in 50 µm microchannel with periodic flow of regular stem cell culture medium.** C17.2 cell adhesion and proliferation was tested in 50 µm tall microchannel with periodic flow of 50, 150, 250, 350, 450 and 550 µL/hour administered every 12 hours. (S2.A–S2.D) Cell morphologies after 1 day of continuous flow of medium at different flow rates. (S2.E) The surface area covered by cells after 1 day of culture is normalized to that after 3 hour of static adhesion (S2.E) to estimate the number of cells in the microchannels. Periodic medium feeding at 250 µL/hour every 12 hours yielded the highest number of adherent cells and significant cell proliferation after 1 day of continuous flow. Scale bar = 100 µm. N≥15.
(TIF)

Figure S3 **Cell adhesion and proliferation under different frequencies.** C17.2 cell adhesion and proliferation was tested under different feeding frequencies in 50 µm tall microchannel with a periodic flow of 250 µL/hour. (S3.A–S3.D) Cell morphologies after 1 day of continuous flow of regular stem cell culture medium at different flow rates. (S3.E) The surface area covered by cells after 1 day of culture was normalized to that after 3 hour of static adhesion to estimate the number of cells in the microchannels. Periodic medium feeding at 250 µL/hour administered every 12 hours yielded the highest number of adherent cells after 1 day of continuous flow. Feeding periods less than 12 hours led to significant less adherent cells in the device. Thus, only feeding periods of 12 hours or higher were used in the work. Scale bar = 100 µm. N≥15.
(TIF)
