# Supplementary figures and images for: Maintenance and Neuronal Cell Differentiation of Neural Stem Cells C17.2 Correlated to Medium Availability Sets Design Criteria in Microfluidic Systems
Source: PLoS One. 2014 Oct 13;9(10):e109815. doi: 10.1371/journal.pone.0109815 (PMC4195690; doi:10.1371/journal.pone.0109815)

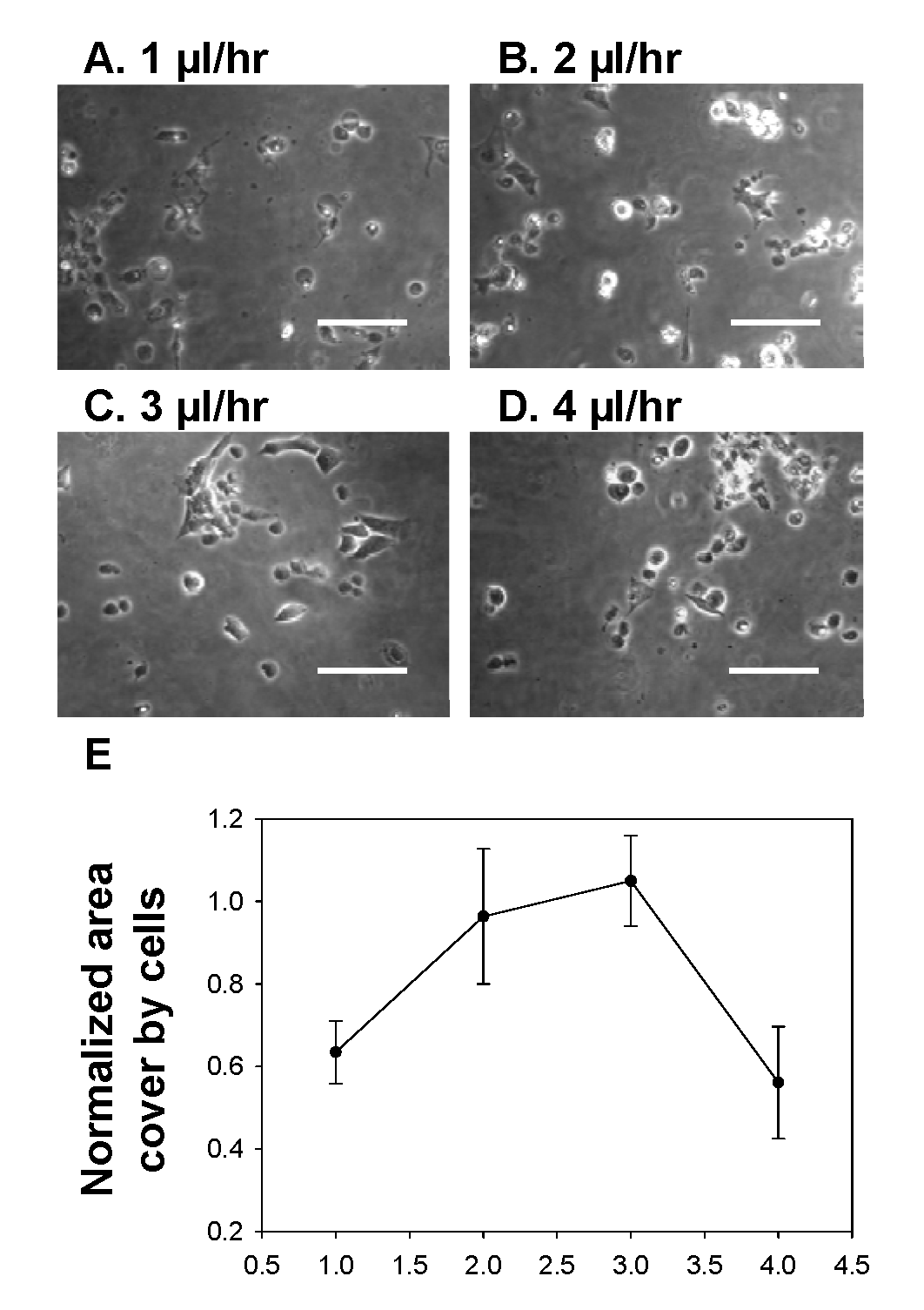

Supplement: Figure S1 — Cell adhesion and proliferation in 50 µm microchannel with continuous flow of regular stem cell culture medium. C17.2 cell adhesion and proliferation was tested in 50 µm tall microchannel with continuous flow of 1, 2, 3 and 4 µL/hour. (S1.A–S1.D) Cell morphologies after 1 day of continuous flow of medium at different flow rates. (S1.E) The surface area covered by cells after 1 day of culture was normalized to that after 3 hour of static adhesion (S1.E) to estimate the number of cells in the microchannels. Continuous medium feeding at 3 µL/hour yielded the highest number of adherent cells after 1 day of continuous flow, but little cell proliferation was observed. Scale bar = 100 µm. N≥15. (TIF) [file pone.0109815.s001.tif]

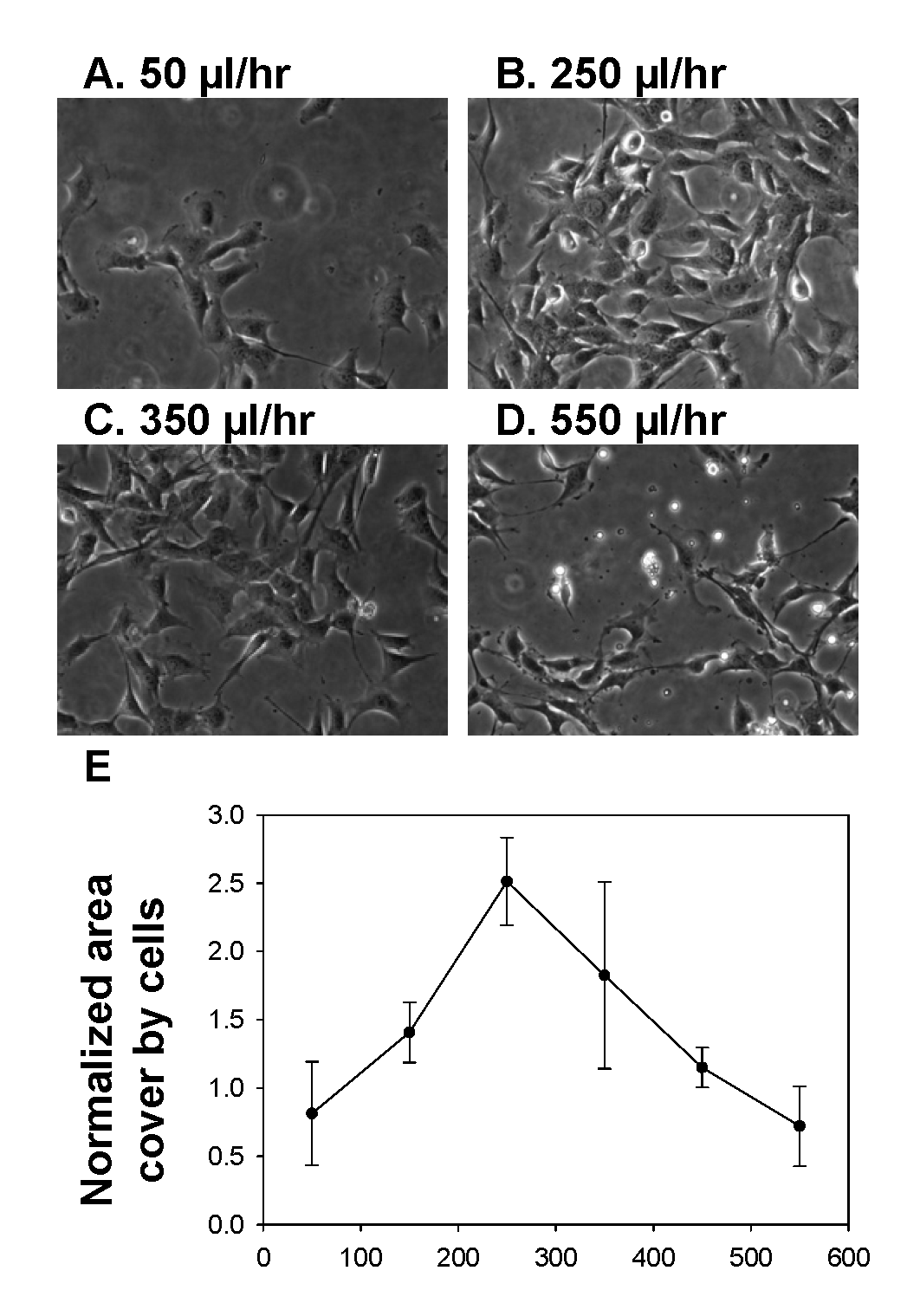

Supplement: Figure S2 — Cell adhesion and proliferation in 50 µm microchannel with periodic flow of regular stem cell culture medium. C17.2 cell adhesion and proliferation was tested in 50 µm tall microchannel with periodic flow of 50, 150, 250, 350, 450 and 550 µL/hour administered every 12 hours. (S2.A–S2.D) Cell morphologies after 1 day of continuous flow of medium at different flow rates. (S2.E) The surface area covered by cells after 1 day of culture is normalized to that after 3 hour of static adhesion (S2.E) to estimate the number of cells in the microchannels. Periodic medium feeding at 250 µL/hour every 12 hours yielded the highest number of adherent cells and significant cell proliferation after 1 day of continuous flow. Scale bar = 100 µm. N≥15. (TIF) [file pone.0109815.s002.tif]

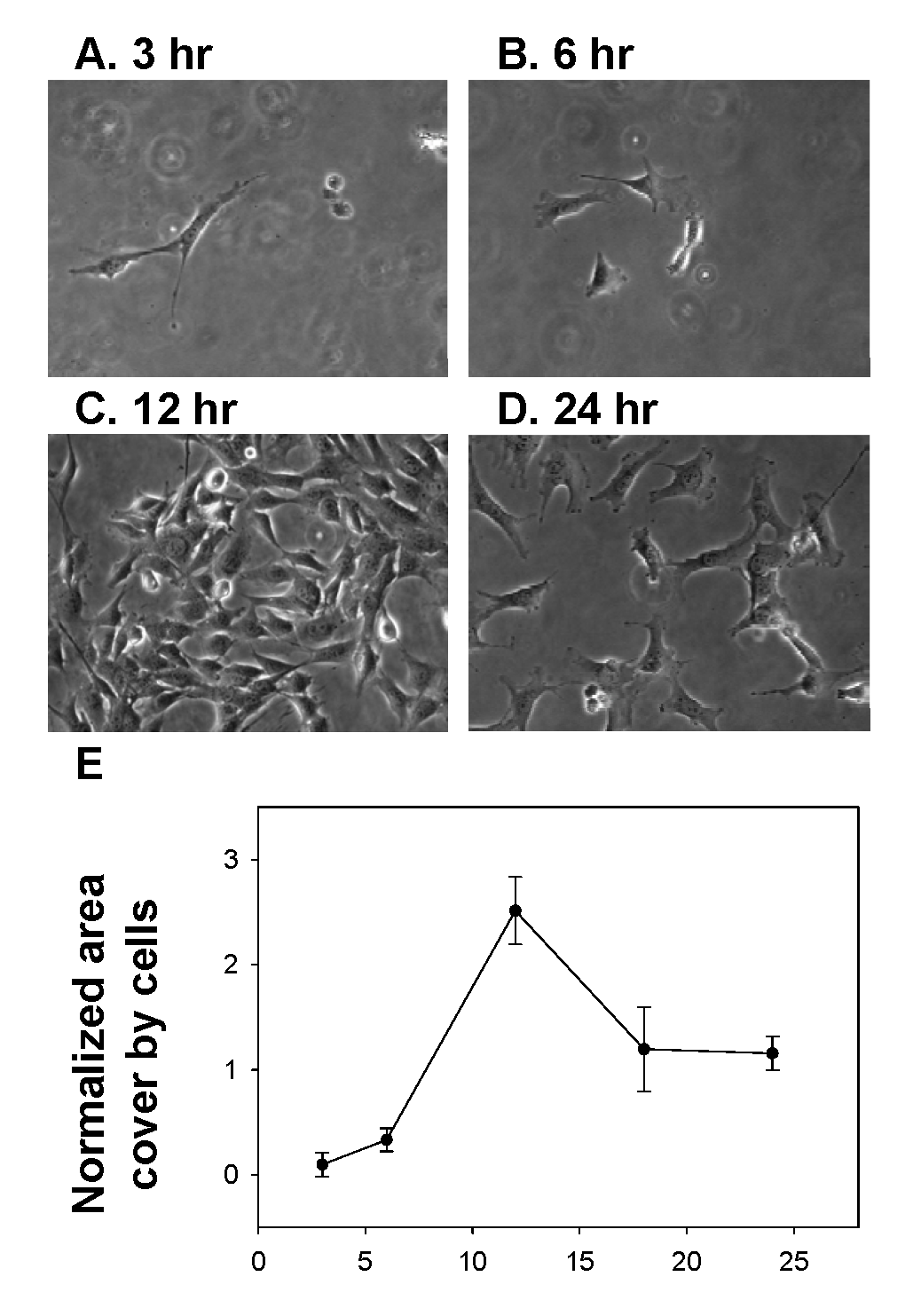

Supplement: Figure S3 — Cell adhesion and proliferation under different frequencies. C17.2 cell adhesion and proliferation was tested under different feeding frequencies in 50 µm tall microchannel with a periodic flow of 250 µL/hour. (S3.A–S3.D) Cell morphologies after 1 day of continuous flow of regular stem cell culture medium at different flow rates. (S3.E) The surface area covered by cells after 1 day of culture was normalized to that after 3 hour of static adhesion to estimate the number of cells in the microchannels. Periodic medium feeding at 250 µL/hour administered every 12 hours yielded the highest number of adherent cells after 1 day of continuous flow. Feeding periods less than 12 hours led to significant less adherent cells in the device. Thus, only feeding periods of 12 hours or higher were used in the work. Scale bar = 100 µm. N≥15. (TIF) [file pone.0109815.s003.tif]
